# Reduced Firing of Nucleus Accumbens Parvalbumin Interneurons Impairs Risk Avoidance in *DISC1* Transgenic Mice

**DOI:** 10.1007/s12264-021-00731-7

**Published:** 2021-06-18

**Authors:** Xinyi Zhou, Bifeng Wu, Wenhao Liu, Qian Xiao, Wei He, Ying Zhou, Pengfei Wei, Xu Zhang, Yue Liu, Jie Wang, Jufang He, Zhigang Zhang, Weidong Li, Liping Wang, Jie Tu

**Affiliations:** 1 Guangdong Provincial Key Laboratory of Brain Connectome and Behavior, CAS Key Laboratory of Brain Connectome and Manipulation, the Brain Cognition and Brain Disease Institute, Shenzhen-Hong Kong Institute of Brain Science-Shenzhen Fundamental Research Institutions, Shenzhen Institute of Advanced Technology, Chinese Academy of Sciences, 518055 Shenzhen, China; 2 University of Chinese of Academy of Sciences, Beijing, 100049 China; 3 Department of Information Technology and Electrical Engineering, Zurich Swiss Federal Institute of Technology Zurich, 8092 Zurich, Switzerland; 4 Department of Biomedical Sciences, City University of Hong Kong, Kowloon Tong, Hong Kong; 5 Virginia Polytechnic Institute and State University, Blacksburg, Virginia 24061 USA; 6 Department of Psychiatry and Behavioral Sciences, Emory University School of Medicine, Atlanta, 30322 USA; 7 Bio-X Institutes, Key Laboratory for the Genetics of Development and Neuropsychiatric Disorders (Ministry of Education), Shanghai Key Laboratory of Psychotic Disorders, and Brain Science and Technology Research Center, Shanghai Jiao Tong University, Shanghai, 200240 China; 8 Center of Brain Science, State Key Laboratory of Magnetic Resonance and Atomic and Molecular Physics, Innovation Academy for Precision Measurement Science and Technology, National Center for Magnetic Resonance in Wuhan, Key Laboratory of Magnetic Resonance in Biological Systems, Wuhan Institute of Physics and Mathematics, Chinese Academy of Sciences, 430071 Wuhan, China; 9 Department of Neuroscience, City University of Hong Kong, Kowloon Tong, Hong Kong; 10 City University of Hong Kong Shenzhen Research Institute, Shenzhen, 518057 China

**Keywords:** *DISC1*, Risk avoidance, Parvalbumin, Nucleus accumbens

## Abstract

A strong animal survival instinct is to approach objects and situations that are of benefit and to avoid risk. In humans, a large proportion of mental disorders are accompanied by impairments in risk avoidance. One of the most important genes involved in mental disorders is disrupted-in-schizophrenia-1 (*DISC1*), and animal models in which this gene has some level of dysfunction show emotion-related impairments. However, it is not known whether *DISC1* mouse models have an impairment in avoiding potential risks. In the present study, we used *DISC1-N* terminal truncation (*DISC1-N*^*TM*^) mice to investigate risk avoidance and found that these mice were impaired in risk avoidance on the elevated plus maze (EPM) and showed reduced social preference in a three-chamber social interaction test. Following EPM tests, c-Fos expression levels indicated that the nucleus accumbens (NAc) was associated with risk-avoidance behavior in *DISC1-N*^*TM*^ mice. In addition, *in vivo* electrophysiological recordings following tamoxifen administration showed that the firing rates of fast-spiking neurons (FS) in the NAc were significantly lower in *DISC1-N*^*TM*^ mice than in wild-type (WT) mice. In addition, *in vitro* patch clamp recording revealed that the frequency of action potentials stimulated by current injection was lower in parvalbumin (PV) neurons in the NAc of *DISC1-N*^*TM*^ mice than in WT controls. The impairment of risk avoidance in *DISC1-N*^*TM*^ mice was rescued using optogenetic tools that activated NAc^PV^ neurons. Finally, inhibition of the activity of NAc^PV^ neurons in PV-Cre mice mimicked the risk-avoidance impairment found in *DISC1-N*^*TM*^ mice during tests on the elevated zero maze. Taken together, our findings confirm an impairment in risk avoidance in *DISC1-N*^*TM*^ mice and suggest that reduced excitability of NAc^PV^ neurons is responsible.

**Supplementary Information:**

The online version contains supplementary material available at 10.1007/s12264-021-00731-7.

## Introduction

Mental disorders are a problem in modern society worldwide, leading to substantial personal and social burdens and yet efficacious therapeutic targets for these disorders are currently lacking [1–3]. The neurobiological mechanisms underlying mental disorders are complicated and are affected by three factors: genes, the environment, and gene-environment interactions [4–6]. The *DISC1* gene is one of the most well-known risk genes used to study the pathophysiology of major mental disorders, such as schizophrenia, bipolar disorder, and major depressive disorder [7]. A study of an extended Scottish family in which a balanced (1; 11) (q42.1; q14.3) chromosomal translocation was found to co-segregate with mental illness led the way to identification of the *DISC1* gene [8]. Many transgenic rodent models have been generated to manipulate the function of this gene [9–11].

In many patients, the mental disorders are accompanied by varying levels of impairment in novelty-seeking [12–14] and risk-avoidance [15–17]. When animals face a novel environment, they show an innate avoidance of risky environments, which can be interpreted as reflecting phenotypes related to anxiety. On the other hand, they exhibit natural exploratory behavior in novel environments. Both exploration of novel environments and risk-avoidance behaviors are conserved across species [18–20], and judgment of the behavioral options related to avoidance or approach is a process necessary for the survival of species and evolutionary fitness. Behavioral tests based on approach-avoidance conflict tasks, such as the elevated plus maze (EPM) are used to test risk-avoidance [21, 22].

Many studies have reported involvement of the nucleus accumbens (NAc) in the regulation and expression of reward, hedonia, and addictive behavior [23–26]. However, an increasing number of studies in both humans and animals have reported NAc involvement in active/inhibitory avoidance [23]. Dopamine receptor type-2 (D2R)-expressing cells in the NAc drive the avoidance of risk [19], and precisely timed phasic stimulation of NAc D2R cells instantaneously converts risk-preferring rats to risk-averse rats [27]. In humans, functional magnetic resonance imaging analyses have shown that hemodynamic changes within the NAc are associated with risk-avoidance behavior [28, 29]. Although studies have confirmed that risk-related behavior is strongly correlated with NAc activity, the neuronal mechanisms underlying this behavior remain poorly understood.

To investigate these mechanisms, we combined *DISC1*-truncated transgenic mice, opto/chemogenetic manipulations, slice electrophysiology, and behavioral analyses to demonstrate that (1) *DISC1-N*^*TM*^ mice have an impairment in risk-avoidance, (2) dysfunction of NAc fast-spiking PV neurons is responsible for the deficits in risk-avoidance, and (3) optogenetic activation of NAc^PV^ neurons rescues the impaired risk-avoidance behavior in *DISC1-N*^*TM*^ mice.

## Materials and Methods

### Animals

We used the following lines: PV-Cre mice (B6; 129P2-Pvalbtm1 (cre) Arbr/J, Jackson Laboratory, stock No.008069), VGAT-ChR2-EYFP mice (B6. Cg-Tg (Slc32a1-COP4*H134R/EYFP) 8Gfng/J, Jackson Laboratory, stock No. 014548), inducible *DISC1-N* terminal fragment transgenic mice (*DISC1-N*^*TM*^, generated in Prof. Weidong Li’s lab, Shanghai Jiao Tong University), and C57BL/6J mice (Guangdong Medical Laboratory Animal Center, Guangzhou, China). To generate the *DISC1-N*^*TM*^ line, we used a pMM-LBDG521R-DISC1-N plasmid containing an a-calmodulin kinase II promoter, a hybrid intron in the 5’ untranslated leader, an HA virus-tag sequence, and a LBDG521R cDNA fused 5’ to the DISC1-N cDNA (encoding protein residues 1-593, the N-terminal portion of the DISC1 protein), as well as a polyadenylation signal. The pMM-LBDG521R-DISC1-N plasmid was digested with SfiI, and transgenic mice were generated by injecting the purified insert into the pronuclei of C57BL/6 zygotes. Founders were back-crossed into C57BL/6J mice. All procedures used were approved by the Animal Research Committee of Shanghai Jiao Tong University. *DISC1-N* spans residues 1–594, which is sufficient for DISC1 nuclear targeting [30] and interacts with glycogen synthase kinase-3 (GSK-3, a serine/threonine protein kinase) [31]. The estrogen receptor ligand-binding domain (ER-LBD) is unable to bind its natural ligand (i.e., estrogen) but is activated specifically by tamoxifen. Without tamoxifen, the transgenic protein is sequestered and degraded by heat-shock chaperone proteins. When tamoxifen binds the ER-LBD, the fusion protein complex undergoes a conformational switch and sets the transgenic protein free. The functional DISC1-N interferes with endogenous DISC1 functions.

Mice were given free access to food pellets and water and were maintained on a 12 h/12 h light/dark cycle (lights on at 08:00). All experiments were approved by the Shenzhen Institutes of Advanced Technology, Chinese Academy of Sciences Research Committee, and all experimental procedures involving animals were carried out in strict accordance with the Research Committee’s animal use guidelines. Surgeries were performed under full anesthesia and every effort was made to minimize animal suffering.

### Behavioral Tests

All behavioral tests were performed blind to mice genotype. Groups of mice were age-matched (8–16 weeks) and, prior to behavioral assays, were handled for 5 min per day for three days to reduce the stress introduced by experimenter contact. All mice were given tamoxifen (Sigma-Aldrich, #T5648) (intraperitoneal injection, 0.05 mg/g, drug concentration: 0.005 g/mL in corn oil) 6 h before behavioral testing unless indicated otherwise. In the oil control group, mice were given the same volume of corn oil (Sigma, C8267-500ML) with no tamoxifen. Behavioral tests using clozapine N-oxide (CNO; MedChemExpress, #HY-17366) were conducted in a 60-min window that began 30 min after CNO administration (intraperitoneal injection, 1 mg/kg). All behavioral tests were recorded using a video camera directly above and videos were analyzed using ANY-maze (Stoelting, USA). The ambient light was approximately 200 lux during all behavior tests.

#### Elevated Plus Maze (EPM)

A plastic EPM was used, consisting of a central platform (5 × 5 cm^2^) with two white open arms (25 × 5 cm^2^) and two white closed arms (25 × 5 cm^2^) with 17-cm-high surrounding walls extending from it to form a plus shape. The maze was 58 cm above the floor. Each mouse was placed in the central area of the EPM with its head facing an open arm and allowed to freely explore for 5 min. The number of entries into each arm and the amount of time spent in each type of arm were recorded.

#### Elevated Zero Maze

The plastic elevated zero maze consisted of an elevated annular platform (external diameter, 50 cm; width, 5 cm) with two opposing enclosed quadrants (height, 15 cm) and two open quadrants. The maze was 80 cm above the floor. Each mouse was placed on the platform with its head facing one of the closed quadrants and its behavior was recorded for 5 min. The number of entries into each quadrant and the amount of time spent in each quadrant were recorded.

#### Three-chamber Social Interaction Test

A three-chambered apparatus (60 × 40 × 25 cm^3^) with a central chamber (20-cm wide) and two side-chambers (each 20-cm wide) was used. Both side-chambers contained an upright empty cylinder made of Plexiglas. The diameter of each cylinder (6 cm) was sufficiently large for a 4-week-old stimulus mouse to easily move around inside. A lid was placed on the top of the cylinder to prevent the test mouse from entering. The cylinder had long vertical fenestrations around its circumference, large enough for a mouse outside the cylinder to easily investigate a mouse inside using vision, audition, and olfaction. During the habituation phase, the test mouse was placed into the center chamber and allowed to freely explore the three chambers for 5 min. Then, in the second phase, the test mouse was gently returned to the central chamber, the two side-chamber entrances were blocked, and then a ‘stranger’ (stimulus mouse) was placed in a cylinder in one side chamber. Both entrances were then opened to allow the test mouse to explore the new environment freely for 5 min. The time spent in each chamber was recorded. All stranger/stimulus mice were of the same gender. To prevent test mice from having a chamber preference unrelated to the stranger mouse, after each experiment, all chambers and cylinders were wiped with alcohol. In addition, the placement of the stranger mouse was alternated between individual experiments to prevent any odor build-up becoming a cue and artificially affecting preference.

### Stereotactic Virus Injection and Optogenetic Manipulation

Adeno-associated viruses (AAVs) carrying Cre-inducible transgenes (AAV-DIO-ChR2-mCherry, titers 3 × 10^12^ particles per mL, AAV-DIO-hM4Di-mCherry, titers 3 × 10^12^ particles per mL, AAV-DIO-mCherry, titers 3 × 10^12^ particles per mL) were packaged in our laboratory. Mice were deeply anesthetized with 1% sodium pentobarbital (Sigma-Aldrich, #P3761, 10 mL/kg body weight, i.p.) and placed in a stereotaxic instrument (RWD Life Science Inc., Shenzhen, China) and the head was fixed. A microinjector pump (UMP3/Micro4, USA) was used, *via* a microliter syringe (10 μL, Hamilton), to inject virus into the target region at 80 nL/min. The needle was left in place for 10 min after the end of injection to avoid reflux of the viral solution. A volume of 200 nL of AAV-DIO-ChR2-mCherry was injected unilaterally into the NAc (AP +1.12 mm, ML +1.50 mm, DV −4.85 mm) for optogenetic and electrophysiological experiments. Similarly, 200 nL of AAV-DIO-hM4Di-mCherry was injected bilaterally into the NAc (AP +1.12 mm, ML ±1.50 mm, DV −4.85 mm) for the experiments on designer receptors exclusively activated by a designer drug (DREADD). In the optogenetic experiments, two weeks after virus injection, mice were implanted with a 200-μm unilateral fiber optic cannula (AP +1.12 mm, ML +1.50 mm, DV −4.65 mm) secured to the skull with denture base material (Shanghai New Century Dental Materials Co., Ltd., China) and dental base acrylic resin powder (An’yang Eagle Brand Dental Materials Co., Ltd., China). The mice were then allowed one week to recover before behavioral experiments began. In the EPM test, blue light stimulation (wavelength, 470 nm; frequency, 20 Hz; width, 5 ms; power, 5–8 mW) was directed through the optical cannula at the NAc during the light-ON stage. The control (mCherry) group underwent the same procedure and received the same intensity of laser stimulation.

### Immunohistochemistry

Each mouse was given an overdose of 1% sodium pentobarbital (15 mL/kg body weight, i.p.) and transcardially perfused with phosphate-buffered saline (PBS) followed by 4% paraformaldehyde (PFA; Aladdin, #C104188) in PBS. The brain was removed and submerged in 4% PFA at 4°C overnight to post-fix, and then transferred to 20% sucrose for one day and then 30% sucrose for 2 days. We used O.C.T. (optimal cutting temperature) compound (Tissue-Tek^®^) to quickly embed the brain before cutting frozen sections. Coronal sections were cut at 30 μm on a cryostat microtome (Lecia CM1950, Germany). The sections were washed with 3 times PBS (3 min, room temperature) to wash out the O.C.T. Then, the sections were immersed in blocking solution (0.3% Triton X-100 and 10% normal goat serum in PBS,) for 1 h at room temperature. The sections were then incubated in primary antiserum (rabbit anti-c-Fos, 1:300, Cell Signaling; rabbit anti-PV, 1:300, Abcam; mouse anti-HA-tag, 1:300, Proteintech) diluted in PBS with 3% normal goat serum and 0.1% Triton X-100 overnight. The following day, the sections were incubated in secondary antibodies at room temperature for 1 h. The secondary antibodies used were Alexa Fluor^®^ 488 or 594-conjugated goat anti-rabbit or anti-mouse IgG antibodies (1:300, Invitrogen, CA, USA). Then the sections were mounted and covers lipped with anti-fade reagent containing DAPI (ProLong Gold Antifade Reagent with DAPI, Life Technologies). The sections were then photographed and analyzed with an Olympus slide scanner (VS120-S6-W) or a Leica TCS SP5 laser scanning confocal microscope. Images were captured and c-Fos staining was manually counted by two experimenters blind to the experimental groups. Definitions of brain regions were based on The Mouse Brain in Stereotaxic Coordinates [32]. Each mouse was sacrificed 1.5 h after the EPM test and the brain was then prepared for c-Fos staining. To compare the difference between c-Fos expression in WT mice and *DISC1-N*^*TM*^ mice within the same brain region, we took the average WT c-Fos expression value as a reference and normalized the data (*N* represents the c-Fos number, *N* (%) represents the normalized data, *n* represents the number of the mouse).

$$N_{{{\text{mean}}}}^{{{\text{WT}}}} = \left( {N_{1}^{{{\text{WT}}}} + N_{2}^{{{\text{WT}}}} + \cdots + N_{n}^{{{\text{WT}}}} } \right)/n;$$

$$N^{{{\text{DISC}}1}}_{{\text{n}}} \left( \% \right) = N^{{{\text{DISC1}}}}_{{\text{n}}} /N^{{{\text{WT}}}}_{{{\text{mean}}}} \times \, 100;$$

$$N^{{{\text{WT}}}}_{{\text{n}}} \left( \% \right) = N^{{{\text{WT}}}}_{{\text{n}}} /N^{{{\text{WT}}}}_{{{\text{mean}}}} \times 100.$$

### Western Blotting

*DISC1-N*^*TM*^ mice and WT littermates were injected with tamoxifen (0.05 mg/g, i.p.) and sacrificed 6 h later. Samples of the hippocampus and NAc of *DISC1-N*^*TM*^ mice and WT littermate controls were homogenized in RIPA (Radio-Immunoprecipitation Assay) protein extraction buffer (Thermo Scientific™, 89900). Supernatants were analyzed using Western blots. The average amount of protein in each electrophoresis channel was ~170 μg. Equal amounts of protein were loaded onto 10% acrylamide gels, followed by transfer and blotting. Anti-mouse HA-tag antibody (1:500, Cell Signaling Technology) was used for immunoblotting.

### Patch-clamp Electrophysiology

For patch-clamp recording, all drugs used were from Sigma-Aldrich unless indicated otherwise. Coronal slices (300 μm) containing the NAc shell (bregma 1.7 to 0.6 mm) were prepared using standard procedures. Brains were quickly removed and chilled in ice-cold modified artificial cerebrospinal fluid (ACSF) containing (in mmol/L): 110 choline chloride, 2.5 KCl, 1.3 NaH_2_PO_4_, 25 NaHCO_3_, 1.3 Na-ascorbate, 0.6 Na-pyruvate, 10 glucose, 2 CaCl_2_, and 1.3 MgCl_2_. Then the NAc slices were cut in ice-cold modified ACSF on a Leica vibroslicer (VT-1200S). The slices were allowed to recover for 30 min in a storage chamber containing regular ACSF at 32–34°C (in mmol/L): 125 NaCl, 2.5 KCl, 1.3 NaH_2_PO_4_, 25NaHCO_3_, 1.3 Na-ascorbate, 0.6 Na-pyruvate, 10 glucose, 2 CaCl_2_, 1.3 MgCl_2_ (pH 7.3–7.4 when saturated with 95% O_2_/5% CO_2_), and thereafter kept at room temperature, until placement in the recording chamber. The osmolarity of all the solutions was 300–320 mOsm/kg. In all electrophysiological experiments, the slices were viewed under an upright microscope (Eclipse FN1, Nikon Instruments) using infrared optics. The recording chamber was continuously perfused with oxygenated ACSF (2 mL/min) at room temperature. Pipettes were pulled on a micropipette puller (Sutter P-2000 Micropipette Puller) with a resistance of 5–10 MΩ. Recordings were made with electrodes filled with intracellular solution (in mmol/L): 130 potassium gluconate, 1 EGTA, 10 NaCl, 10 HEPES, 2 MgCl_2_, 0.133 CaCl_2_, 3.5 Mg-ATP, 1 Na-GTP. Action potential firing frequency was analyzed in current-clamp mode in response to a 2-s depolarizing current step. All recordings were conducted with a MultiClamp700B amplifier (Molecular Devices). Currents were low-pass filtered at 2 kHz and digitized at 20 kHz using an Axon Digidata 1440A data acquisition system and pClamp 10 software (both from Molecular Devices). Series resistance (Rs) was 10–30 MΩ and regularly monitored throughout the recordings. Data were discarded if Rs changed by >30% over the course of data acquisition.

### *In Vivo* Electrophysiological Recordings

Anesthesia was induced in all mice with 2–3% isoflurane (RWD Life Science Inc., Shenzhen, China) and then maintained at 1%–2%. Body temperature was maintained at 36–37°C using a heating pad. Each mouse was secured in a stereotaxic apparatus before a midline incision was made and the skull exposed. A titanium plate was then implanted above the skull with the target brain area in the recording window. After 3 days, mice were habituated to a head-fixation apparatus for 0.5 h per day for 5 days. Mice were then injected with tamoxifen 6 h prior to recording, and then anesthetized with isoflurane to remove the dura. After surgery, each mouse was returned to its home cage to recover. A 4-shank silicon probe with 64 channels (Lotus Biochips) was then implanted and aimed at the target region (1.35 mm anterior to bregma; 0.6 mm lateral to the midline; and 3.5–4.5 mm depth) with a micromanipulator (Scientifica, U.K). The 64-channel electrode was connected to two 32-channel head-stage amplifiers and then to a 256-channel recording device (Intan, U.S.A). Three recording sessions (10 min/session) were conducted 6 h after tamoxifen injection using a sampling rate of 30 kHz. The mice were awake during the recordings. After *in vivo* recording, the recording sites were marked with electrolytic lesions and the animals were deeply anesthetized with 10% chloral hydrate (0.4 mg/kg) and transcardially perfused with PBS followed by 4% PFA (wt/vol). Each brain was dissected and post-fixed at 4°C in 4% PFA overnight. Frozen sections of the whole area were cut at 40 μm, and the recording locations were reconstructed.

### Single-unit Spike Sorting and Data Analysis

Single-unit spike sorting was performed using Kilosort2 [33] and the similarity of each unit to all other units were calculated for each data set. Pre-sorted results were manually checked with Phy2 (https://github.com/cortex-lab/phy). Of 298 units, 131 were from *DISC1-N*^*TM*^ mice and 167 from WT mice. Units with high similarity (equal to 1.000) and the same waveform pattern were combined using Phy2. To separate putative fast-spiking neurons from putative non-fast-spiking neurons, two waveform features, valley-to-peak time and half-peak width, were individually calculated [34–36]. Based on these two features, eight FS neurons were found from 131 *DISC1-N*^*TM*^ neurons and 15 FS neurons from 167 WT neurons. MatLab 2020a (MathWorks) was used for all analyses including waveform feature analyses and spike sorting.

## Results

### ***DISC1-N***^***TM***^ Mice Have Impaired Risk-avoidance Behavior

**Fig. 1:**
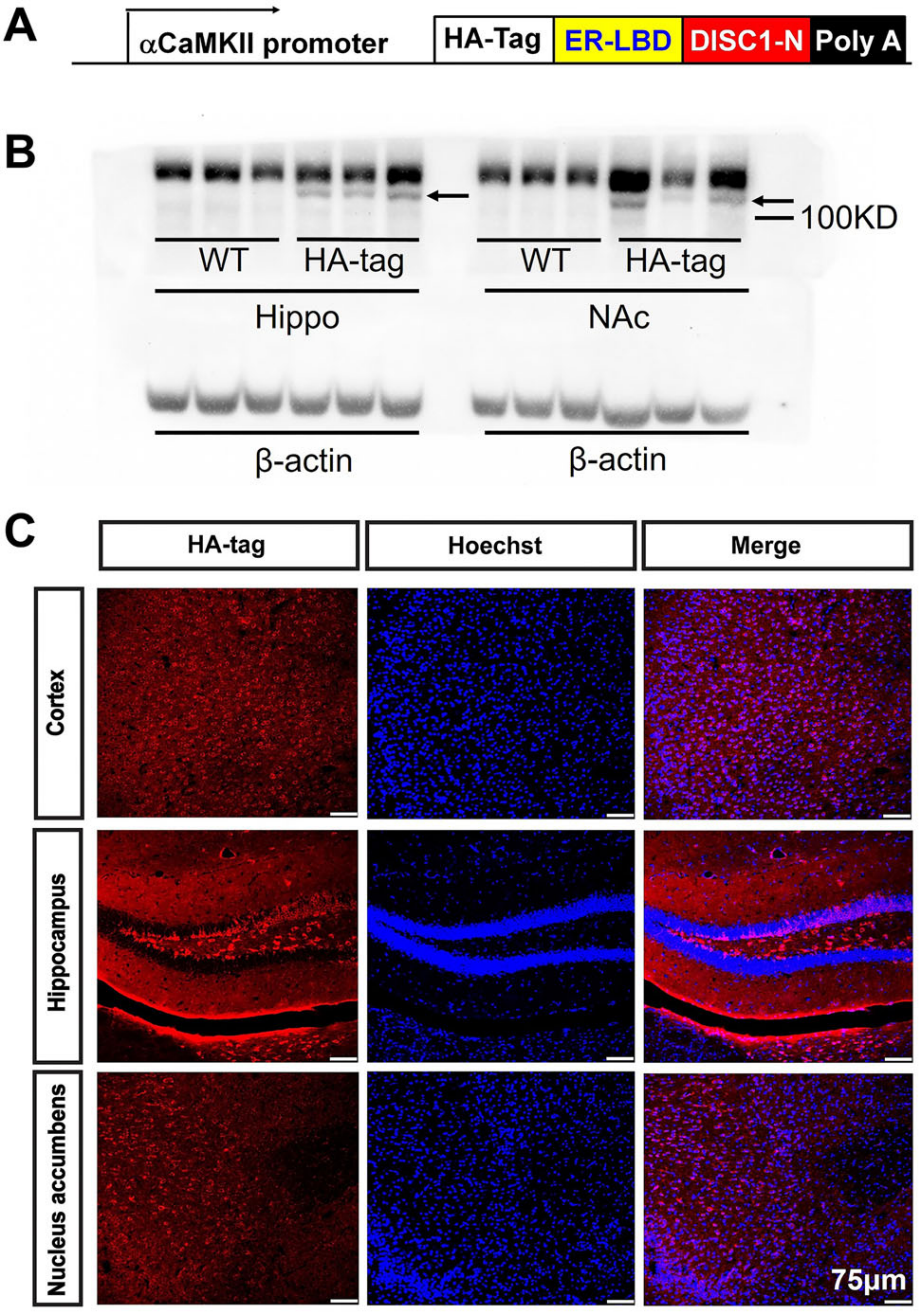
Deriving transgenic mice with the inducible DISC1-N terminal fragment **A** Schematic showing the inserted expressed sequence in *DISC1-N*^*TM*^ mice. **B** Western blots showing the expression of the DISC1-N truncated protein by HA-tag in the hippocampus (Hip) and nucleus accumbens (NAc) of transgenic (*DISC1-N*^*TM*^) but not in WT mice (arrows indicate the DISC1-N protein). **C** Staining for HA-tag (red) and Hoechst 33258 (blue) in the cortex, hippocampus, and NAc of *DISC1-N*^*TM*^ mice.

The *DISC1* transgenic mouse strain used was an N-terminal fragment isoform under an *CamkIIa* promoter (Fig. 1A). Intraperitoneal injection of tamoxifen leads to the inducible expression of functional DISC1-N, which interferes with endogenous DISC1 functions. To verify the expression of the inserted gene, mice were sacrificed 6 h after tamoxifen injection and their brains were removed for western blotting and staining. The protein expression of DISC1 in the hippocampus and NAc was determined (Fig. 1B). The HA-tag was co-stained with DNA-specific fluorescent Hoechst 33258 in three brain areas: the cortex, the hippocampus, and the NAc (Fig. 1C). These data revealed that the *DISC1-N* truncation was widely expressed in the transgenic mice line.

**Fig. 2:**
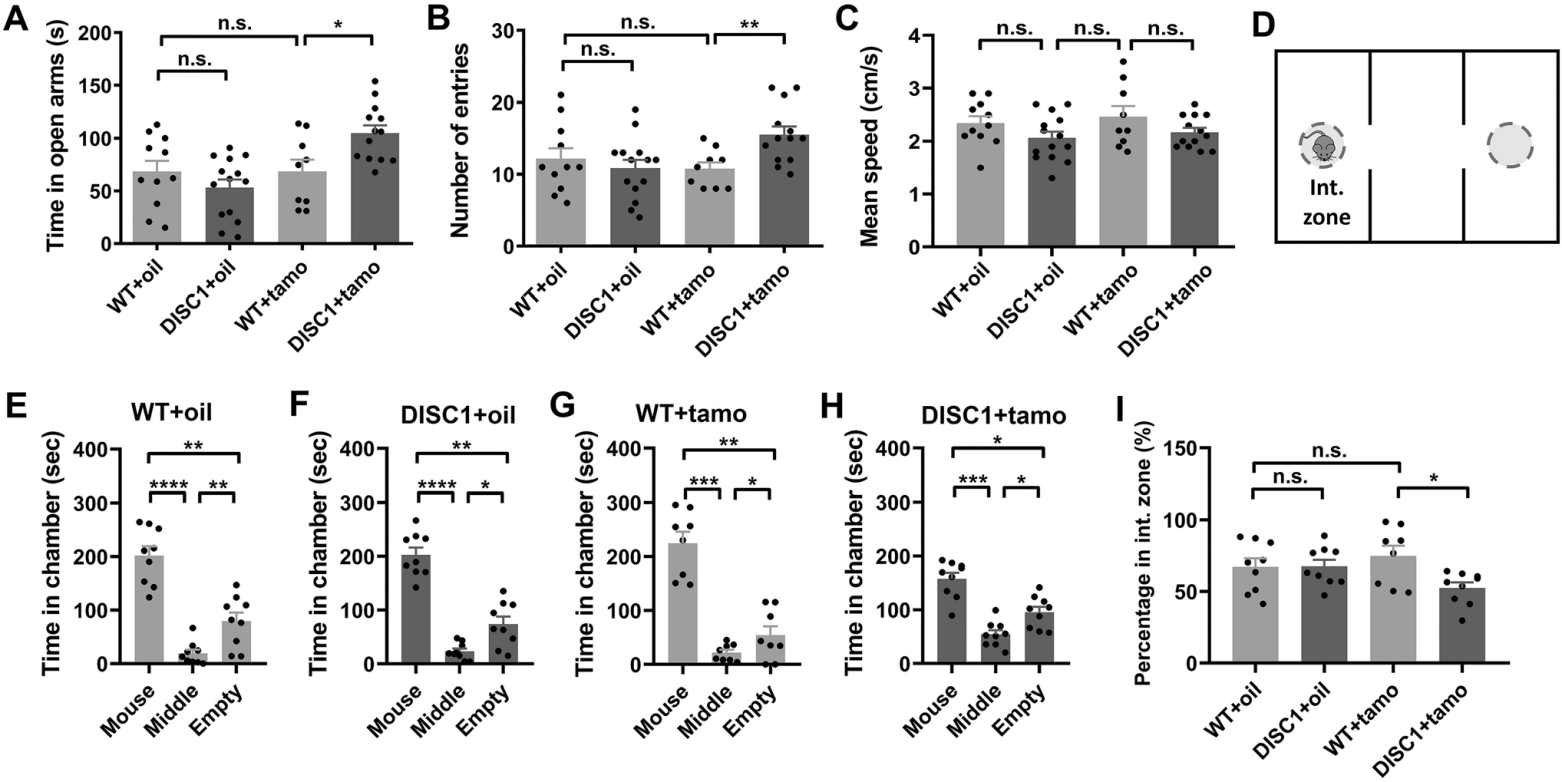
*DISC1-N*^*TM*^ mice are impaired in risk-avoidance. **A–C** Time spent in the open arms (**A**), number of entries into the open arms (**B**), and mean speed (**C**) during the EPM test (unpaired *t*-test, *P = 0.0105, **P = 0.0058; *n* = 11, 14, 9, and 13 from left to right). **D** Diagram of the three-chamber social interaction task (Int. zone is the interaction zone). **E–H** Time spent in the chamber during the three-chamber social interaction task in the WT + oil group (paired *t*-test, upper **P = 0.0058, and lower **P = 0.0078, ****P <0.0001; *n* = 9), the DISC1 + oil group (*P = 0.0128, and **P = 0.0014, ****P <0.0001; *n* = 9), the WT + tamo group (*P = 0.0309, and **P = 0.003, ***P =0.0001; *n* = 8) and the DISC1 + tamo group *P = 0.0103, and *P = 0.0234, ***P = 0.0003; *n* = 9). **I** Percentage of time spent in the interaction chamber during the three-chamber social interaction task (*P = 0.0131; *n* = 9, 9, 8, and 9 from left to right).

We next asked whether expression of the N-terminal fragment isoform of the *DISC1* gene affected risk-avoidance behavior in the EPM. Six hours after tamoxifen administration (i.p.), *DISC1-N*^*TM*^ mice spent significantly more time in the open arms and made more entries to the open arms than control groups, including the WT with tamoxifen (WT + tamo), WT with vehicle (WT + oil), and DISC1 with vehicle (DISC1 + oil) groups (Fig. 2A, B). There was no significant difference between the four groups in mean speed (Fig. 2C). Furthermore, there was no difference between the DISC1 + tamo and WT + tamo groups in speed in the open or closed arms. Neither was there a difference in the total distance traveled, or time spent in the center (Table 1). Therefore, the difference in time spent in the open arms and the number of entries into the open arms were unlikely to have been confounded by changes in locomotion or by intraperitoneal administration. These results suggest that the expression of the N-terminal fragment isoform of the *DISC1* gene influences risk-avoidance behavior in the EPM test.

**Table 1:** Results of the elevated plus-maze test. Values are presented as the mean ± SEM (unpaired *t*-test, *P = 0.0105, **P = 0.0037, and 0.0058 from top to bottom).

	WT + tamo	DISC1 + tamo
Entries into closed arms	16.44 ± 1.709	14.54 ± 1.352
Time spent in closed arms (s)	162.3 ± 9.797	124.8 ± 6.681**
Velocity in closed arms (cm/s)	2.976 ± 0.2407	2.791 ± 0.1366
Entries into open arms	10.78 ± 0.8941	15.54 ± 1.119**
Time spent in open arms (s)	68.53 ± 11.20	104.9 ± 7.416 *
Velocity in open arms (cm/s)	2.001 ± 0.2575	1.962 ± 0.09793
Total distance traveled (m)	7.419 ± 0.5981	6.527 ± 0.2433
Time spent in center (s)	71.21 ± 5.819	70.38 ± 5.791

Given that some *DISC1*-mutant mouse strains also show deficits in social behavior [37], we used a three-chamber social interaction test to determine sociability in *DISC1-N*^*TM*^ mice. Mice normally prefer to spend more time with other mice [38]. In all four groups, mice had a preference for the chamber with the stranger mouse rather than the empty chamber (Fig. 2 E–H). However, following tamoxifen injections, the percentage of time that *DISC1-N*^*TM*^ mice spent with the stranger mouse was less than the WT + tamo control group (Fig. 2I). Although the *DISC1-N*^*TM*^ mice displayed a preference for the stranger mouse, the time spent interacting with the stranger mouse was less than with the WT mice.

### In ***DISC1-N***^***TM***^ Mice, c-Fos Staining Indicates that the NAc Plays a Role in Abnormal Risk-avoidance Behavior

**Fig. 3:**
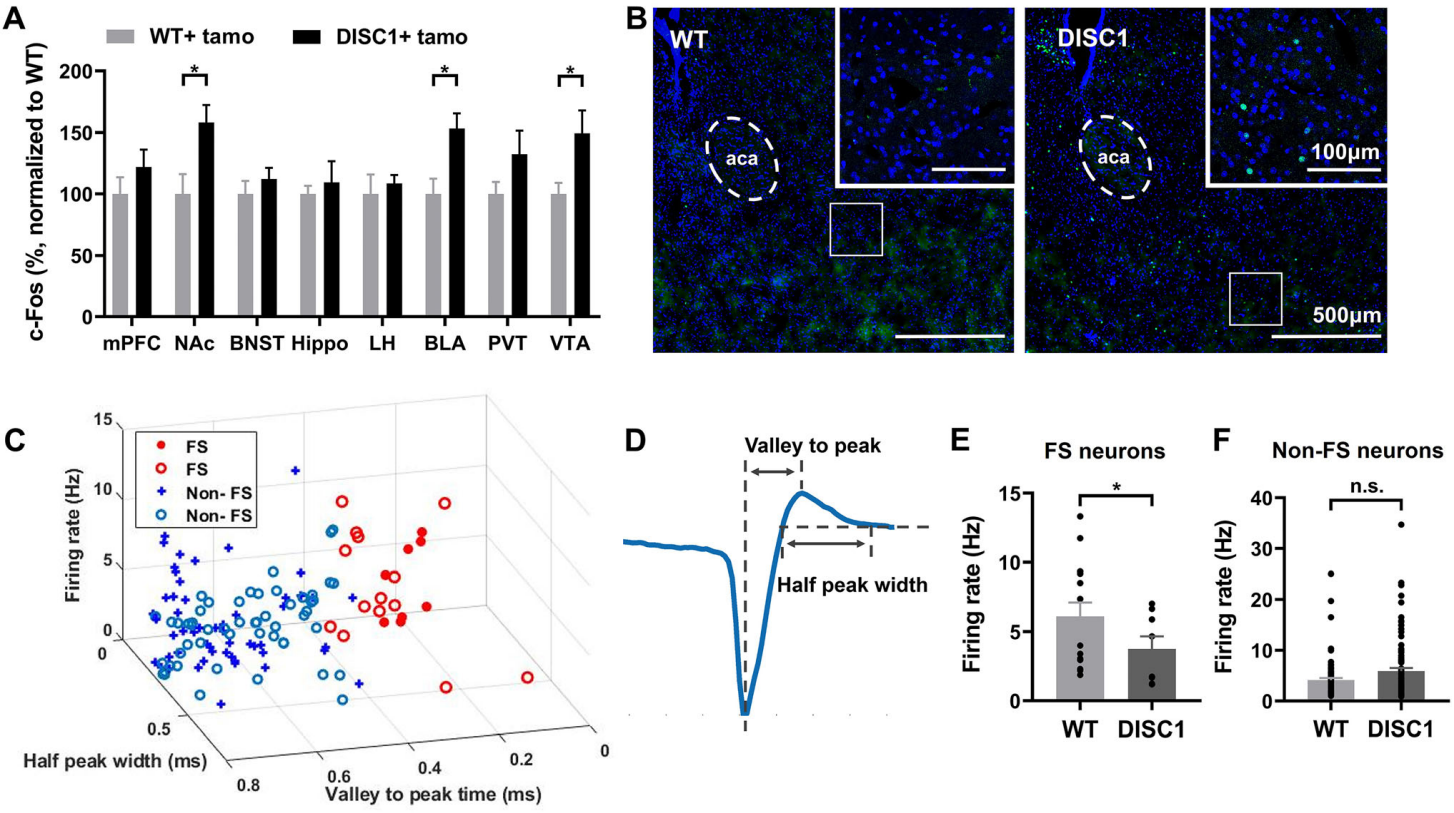
Both c-Fos expression and *in vivo* electrophysiological recordings suggest that NAc participates in the risk-avoidance impairment in *DISC1-N*^*TM*^ mice. **A** Comparison of c-Fos expression in WT and *DISC1-N*^*TM*^ mice after the EPM test (unpaired *t*-test, *P = 0.0215, 0.0123, and 0.0368 from left to right; *n* = 6/group). **B** Representative staining for c-Fos expression in the NAc after the EPM task. **C** Scatter-plot showing the firing rate, half peak width, and valley-to-peak time for *DISC1-N*^*TM*^ mice and WT mice (131 units from 2 *DISC1-N*^*TM*^ mice and 167 units from 2 WT mice; filled circles and plus signs represent *DISC1-N*^*TM*^ mice, open circles represent WT mice). **D** Schematic of an action potential waveform showing the half-peak width and valley-to-peak. **E** Comparison of the firing rate of putative FS neurons between *DISC1-N*^*TM*^ mice and WT mice after tamoxifen injection (unpaired *t-*test, *P = 0.0371; *n* = 15 left, *n* = 8 right). **F** Firing rate comparison of putative non-FS neurons between *DISC1-N*^*TM*^ mice and WT mice after tamoxifen injection (unpaired *t*-test; *n* = 88 left, *n* = 96 right).

After finding an impairment in risk avoidance in *DISC1-N*^*TM*^ mice, we then aimed to determine which brain area is involved. Following EPM testing, we performed c-Fos staining in 8 emotion-related brain areas: the medial prefrontal cortex, the NAc, the bed nucleus of the stria terminalis (BNST), the hippocampus, the lateral hypothalamus, the basolateral amygdala (BLA), the paraventricular nucleus of the thalamus, and the ventral tegmental area (VTA). Both *DISC1-N*^*TM*^ mice and WT mice were given tamoxifen 6 h before the EPM test. We found that c-Fos expression in the NAc, BLA, and VTA was significantly higher in *DISC1-N*^*TM*^ mice than in WT mice (Fig. 3A). Our previous work showed that neurons in the NAc can control behavioral states and regulate mouse performance in the EPM test [39]. Moreover, NAc involvement in active/inhibitory avoidance has been reported [23]. Thus, we hypothesized that the NAc plays a role in abnormal risk-avoidance behavior in *DISC1-N*^*TM*^ mice.

### ***In Vivo*** Electrophysiological Recordings from FS Neurons in ***DISC1-N***^***TM***^ Mice Show Reduced Firing Rates after Tamoxifen Injection,

To further investigate which cell-types in the NAc participate in the risk-avoidance impairment in *DISC1-N*^*TM*^ mice, we conducted *in vivo* single-cell recording in the NAc of *DISC1-N*^*TM*^ and WT mice 6 h after tamoxifen injection. Distinct sub-types of NAc neurons were classified based on their major electrophysiological properties [34–36]. Neurons were classified as putative FS neurons and putative non-FS neurons based on the valley-to-peak time and half-peak width (Fig. 3C, D). The average firing rates of the FS neurons was lower in *DISC1-N*^*TM*^ mice than in WT mice (Fig. 3E), while there was no difference between these groups for non-FS neurons (Fig. 3F). There are reports of PV interneurons, which are FS neurons, in the NAc [40, 41] and our previous work has shown that PV interneurons in the NAc regulate the performance of chronically-stressed mice in the EPM [39]. Thus, we propose that PV interneurons in the NAc are responsible for the abnormal risk-avoidance behavior of *DISC1-N*^*TM*^ mice.

### PV Neurons in the NAc are Less Excitable in ***DISC1-N***^***TM***^ Mice

**Fig. 4:**
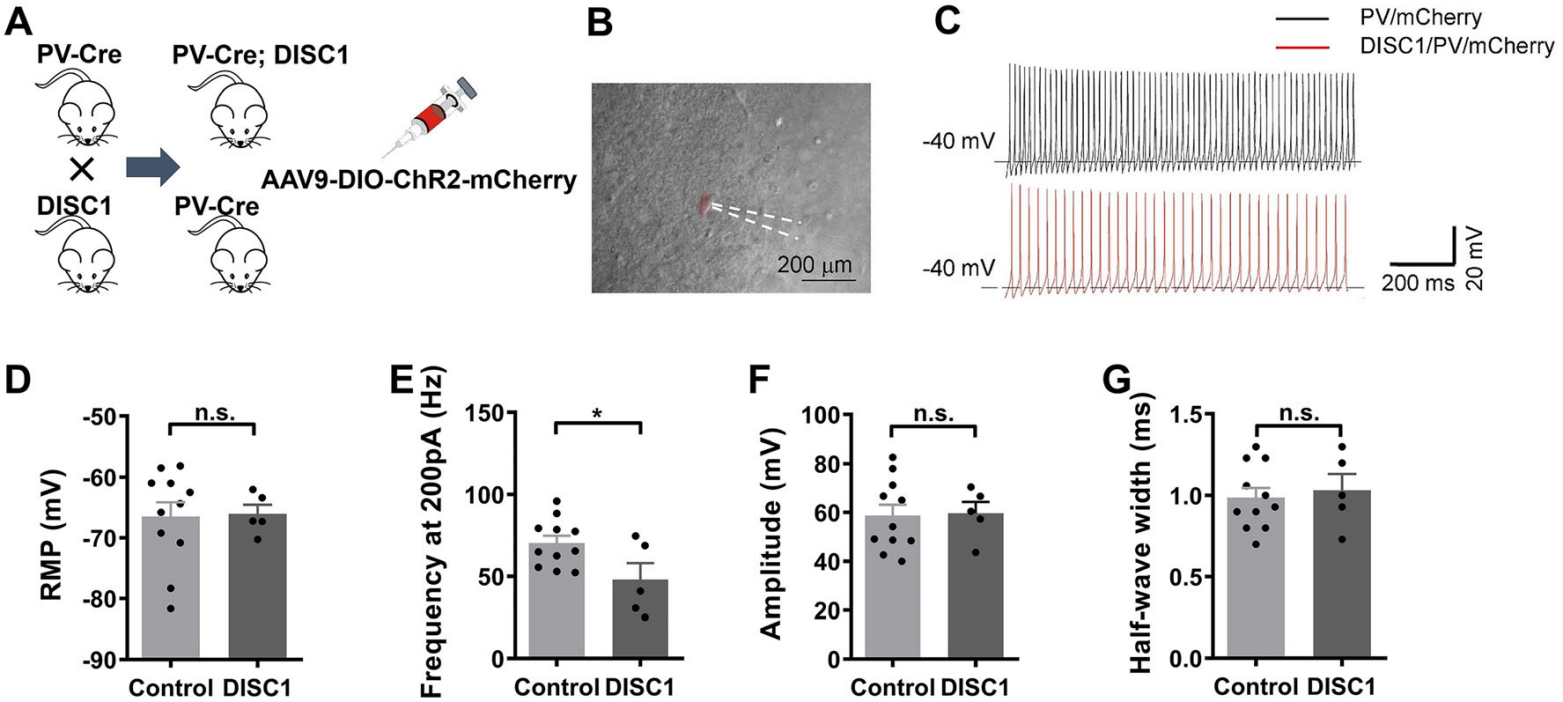
Recordings in acute brain slices from PV-Cre/*DISC1-N*^*TM*^ and PV-Cre mice. **A** Cartoon showing the transgenic mice used. **B** Representative photomicrograph showing patch recording on a PV neuron. **C** Representative action potentials stimulated by 200-pA current. **D** Resting membrane potential of PV neurons (*n* = 11 left, *n* = 5 right). **E–G** Frequency (**E**), amplitude (**F**), and half-wave width (**G**) of action potentials during 200-pA current stimulation (unpaired *t*-test, *P = 0.0308; *n* = 11 left, *n* = 5 right).

To further explore PV function in *DISC1-N*^*TM*^ mice, we investigated the electrophysiological characteristics of NAc^PV^ neurons using whole-cell patch-clamp recordings. We used PV-Cre and *DISC1-N* truncated double-transgenic mice and stereotaxically injected AAV-DIO-ChR2-mCherry into the NAc. In a PV-Cre control group, AAV-DIO-ChR2-mCherry was stereotaxically injected into the NAc area. After waiting 3–4 weeks for full expression of the virus, we prepared acute brain slices 6 h after tamoxifen injection (both in double-transgenic and control mice) and recorded from PV neurons under the current-clamp model. Fluorescent mCherry, carried by the virus, enabled identification of PV neurons under the microscope (Fig. 4B).

We delivered different step-current stimuli to the PV neurons at 100, 150, and 200 pA. Only the 200-pA current was able to induce stable action potentials (APs) in all PV neurons. We compared the firing rates in the control and *DISC1-N*^*TM*^ groups using 200-pA current stimulation and found that PV neurons in *DISC1-N*^*TM*^ mice had significantly lower firing rates than those in the PV-Cre control mice (Fig. 4C, E). This indicated that NAc^PV^ neurons are less excitable in *DISC1-N*^*TM*^ mice than in control mice and that they may be responsible for the impairment in risk-avoidance behavior in *DISC1-N*^*TM*^ mice. There was no difference in the resting membrane potential (Fig. 4D) or amplitude and half-wave width of APs evoked by a 200-pA current (Fig. 4F, G) between NAc^PV^ neurons in *DISC1-N*^*TM*^ and PV-Cre control mice.

### Optogenetic Activation of PV Neurons in the NAc Rescues the Risk-avoidance Impairment in ***DISC1-N***^***TM***^ Mice

**Fig. 5:**
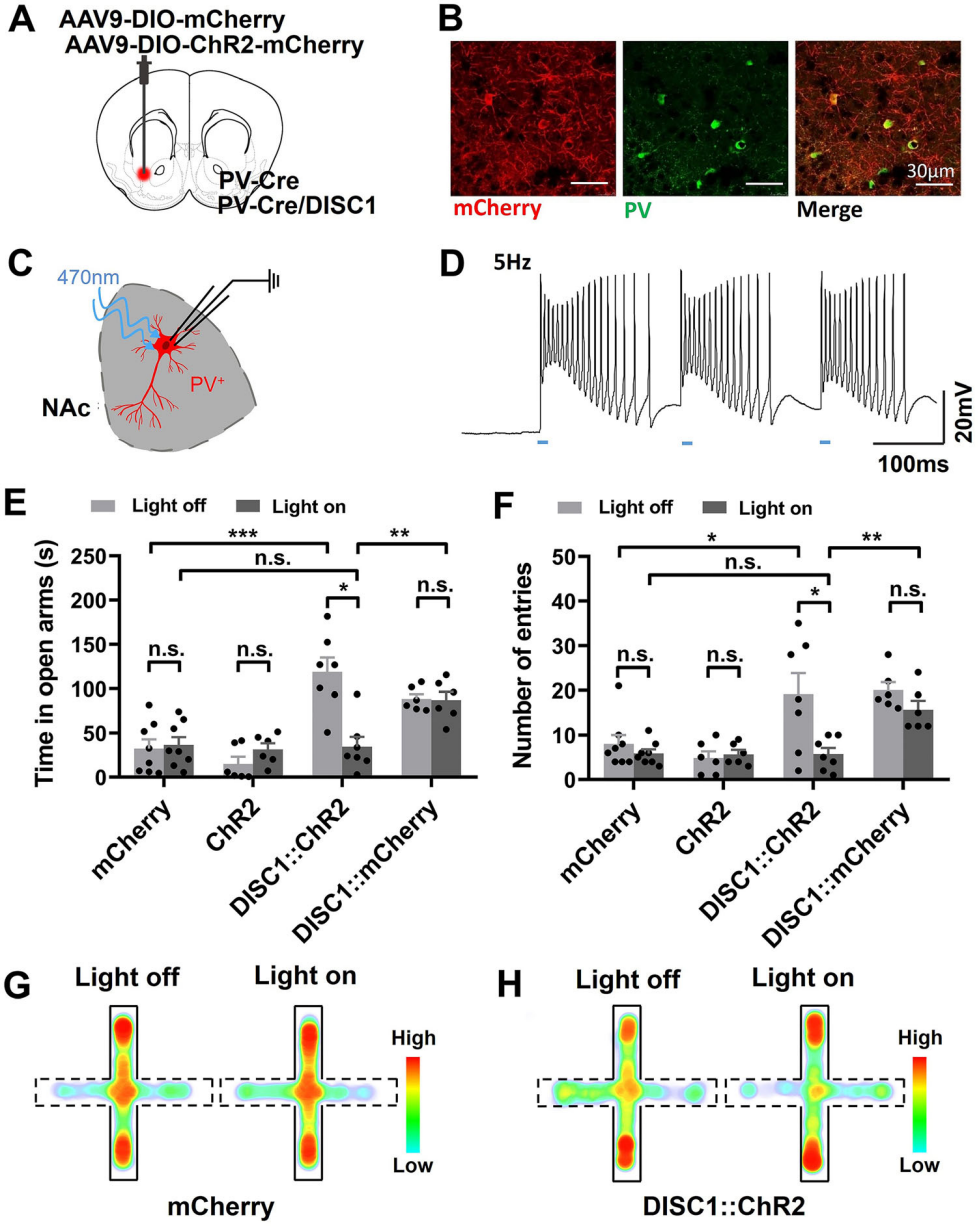
Optogenetic stimulation of NAc^PV^ neurons rescues the risk-avoidance deficiency in *DISC1-N*^*TM*^ mice. **A** Cartoon showing the injection of virus. **B** Neurons in the NAc infected with AAV-DIO-ChR2-mCherry (red) co-stained with PV neurons (green). **C** Cartoon showing patch-clamp recording from a PV neuron in an acute brain slice. **D** Example recording of light-evoked action potentials in NAc PV neurons. **E, F** Time spent in the open arms (**E**) and number of entries into the open arms (**F**) before and after blue light (470 nm) stimulation during the EPM test (Left: paired *t*-test, *P = 0.0121, unpaired *t*-test, **P = 0.0047, ***P = 0.0005.Right: paired *t*-test, upper *P = 0.0404, unpaired *t*-test, lower *P = 0.0401, **P = 0.0016; *n* = 8, 6, 7, and 6 from left to right). **G, H** Average heatmaps illustrating the time spent by a PV-Cre mouse in the open and closed arms in the EPM (**G**) and a PV-Cre/ *DISC1-N*^*TM*^ mouse (**H**) before and after blue light (470 nm) stimulation (dotted lines represent the open arms).

The results above indicate that NAc^PV^ neurons are dysfunctional in *DISC1-N*^*TM*^ mice. Next, we tested whether modulation of PV neurons in the NAc rescues the risk-avoidance impairment in *DISC1-N*^*TM*^ mice. We selectively activated NAc^PV^ neurons by delivering blue light (5 ms per pulse, 60 Hz) to the NAc in PV-Cre and *DISC1-N* truncated double-transgenic mice unilaterally infected in the NAc with AAV-DIO-ChR2-mCherry (Fig. 5A, B). PV-Cre mice unilaterally infected in the NAc with AAV-DIO-mCherry or AAV-DIO-ChR2-mCherry were also used as control groups (Fig. 5A). The function of ChR2-expressing PV neurons was verified using whole-cell patch-clamp recordings in slices containing the NAc area from PV-Cre mice (Fig. 5C, D). EPM tests were conducted 6 h after tamoxifen injection. During blue light stimulation, *DISC1-N*^*TM*^ mice not only spent significantly less time in the open arms but also had fewer entries into the open arms than in the light-OFF phase (Fig. 5E, F). Furthermore, in the light-ON phase, there was no difference between ChR2-expressing *DISC1-N*^*TM*^ mice and non-ChR2-expressing PV-Cre control mice in the time spent in the open arms, or in the number of entries into the open arms (Fig. 5E, F). On the other hand, when comparing the light-ON phase with the light-OFF phase, there was no difference between the time spent in the open arms and the number of entries into the open arms in the non-ChR2-expressing *DISC1-N*^*TM*^ control group (Fig. 5E, F). These results suggest that PV neurons in the NAc can rescue the abnormal risk-avoidance behavior in the EPM task shown by *DISC1-N*^*TM*^ mice. Regarding the ChR2-expressing PV-Cre control group, when stimulated with blue light, the time spent in the open arms and the number of entries into the open arms did not differ from the no-light state (Fig. 5E, F). Activation of NAc^PV^ neurons in mice with normal *DISC1* gene expression did not significantly influence behavior. This indicates that in normal mice, PV neurons play a role in maintaining an appropriate risk-avoidance state.

Of the neurons in the NAc, 95% are medium spiny GABAergic neurons (MSNs). We tested whether the activation of GABAergic neurons in the NAc could rescue the abnormal risk-avoidance behavior in *DISC1-N*^*TM*^ mice. By crossing Vgat-ChR2 mice with *DISC1-N* truncation transgenic mice, we labeled GABAergic neurons with ChR2 (Fig. S1A, B). Following tamoxifen injection, *DISC1*/Vgat-ChR2 double-transgenic mice spent more time in the open arms and had more open-arm entries than WT littermates (Fig. S1C, D). Photostimulation of the NAc at 60 Hz led to a significant reduction in time spent in the open arms, and the number of entries into the open arms in *DISC1*/Vgat-ChR2 double-transgenic mice, while compared to WT littermates, specific optogenetic activation of NAc^GABA^ did not fully rescue the abnormal risk-avoidance behavior in *DISC1*^*TM*^ mice (Fig. S1C, D). This result further confirms that NAc^PV^ neurons play an important role in risk-avoidance behavior in *DISC1-N*^*TM*^ mice during the EPM task.

### Chemogenetic Inhibition of NAc PV Neurons Mimics the Abnormal Risk-avoidance Behavior in ***DISC1-N***^***TM***^ Mice

**Fig. 6:**
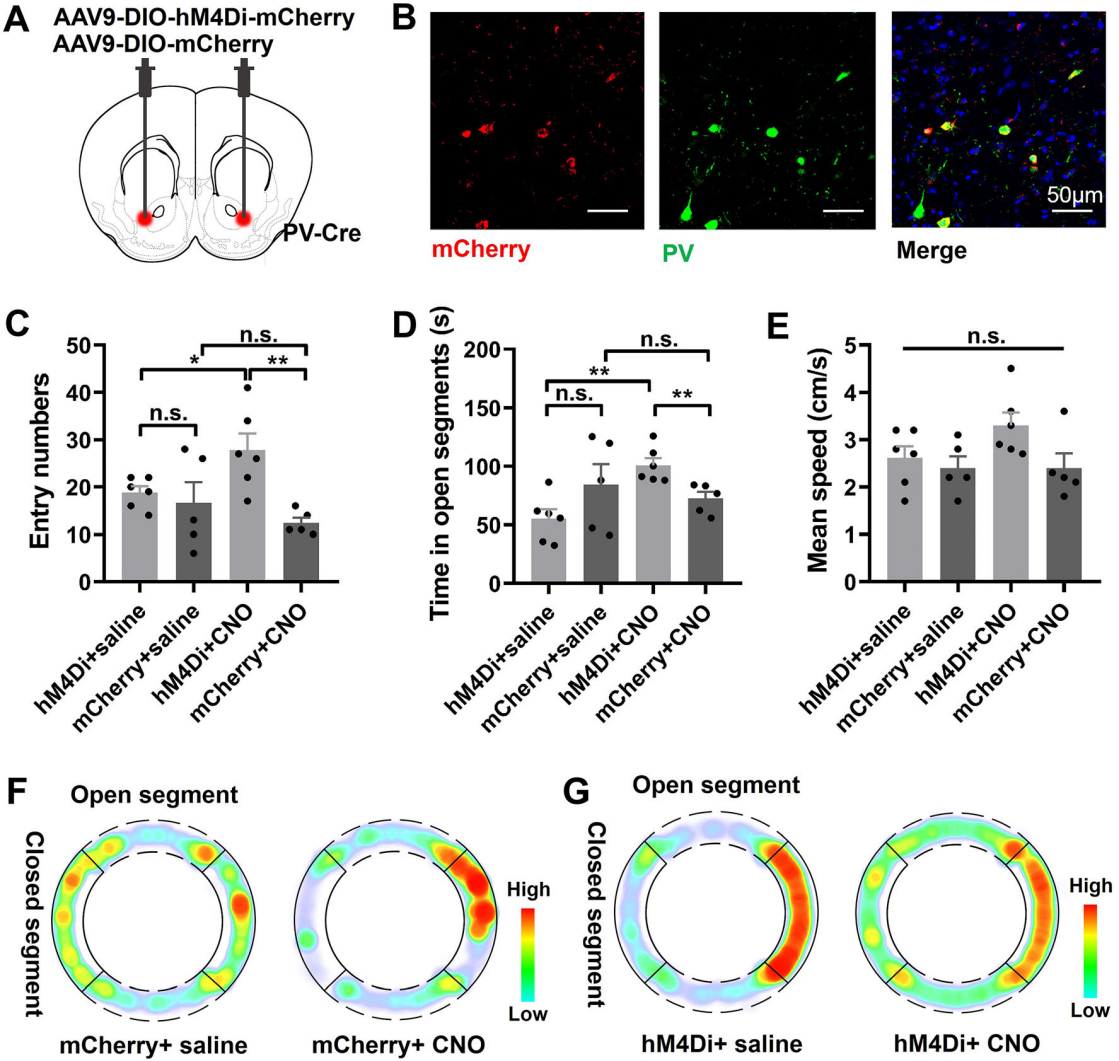
Inhibition of PV neuronal activity in the NAc mimics DISC1-like abnormal risk-avoidance behavior. **A** Cartoon of the injection of virus. **B** Neurons in the NAc infected with AAV-DIO-hM4Di-mCherry (red) co-stained with PV neurons (green). **C** Number of entries into the open segments before and after CNO administration during the elevated zero maze (EOM) test (hM4Di + saline *vs* hM4Di + CNO, mCherry + saline *vs* mCherry + CNO, paired *t*-test, *P = 0.0297; hM4Di + CNO *vs* mCherry + CNO, mCherry + saline *vs* hM4Di+saline, unpaired *t*-test, **P = 0.0038; *n* = 6 hM4Di group, *n* = 5 mCherry group). **D** Time spent in the open segments before and after CNO administration during the EOM test (hM4Di + saline *vs* hM4Di + CNO, mCherry + saline *vs* mCherry+CNO, paired *t*-test, **P = 0.0074; hM4Di + CNO *vs* mCherry + CNO, mCherry + saline *vs* hM4Di + saline, unpaired *t*-test, **P = 0.0087; *n* = 6 hM4Di group, *n* = 5 mCherry group). **E** Mean speed before and after CNO administration during the EOM test (hM4Di + saline *vs* hM4Di + CNO, mCherry + saline *vs* mCherry + CNO, paired *t*-test; mCherry + saline *vs* hM4Di + saline, hM4Di + CNO *vs* mCherry + CNO, unpaired *t*-test; *n* = 6 hM4Di group, *n* = 5 mCherry group:). **F–G** Average heatmaps showing the time spent in each location in the EOM test before and after CNO administration.

We found that modulation of PV neurons in the NAc rescued the abnormal risk-avoidance behavior in *DISC1-N*^*TM*^ mice. To assess the contribution of these neurons to the regulation of the abnormal risk-avoidance behavior, we used a DREADD to inhibit the activity of NAc PV neurons in mice with normal DISC1 function. PV-Cre mice were bilaterally infected with AAV9-DIO-hM4Di-mCherry in the NAc (treatment group) or AAV9-DIO-mCherry (control group) (Fig. 6A, B). After three weeks of recovery, risk avoidance behavior was tested on an elevated zero maze without CNO injection. Then, after a one-week interval, the same group of mice were given CNO and tested again on the elevated zero maze. We found that, without CNO injection, there was no difference between the treatment and control groups in either the number of entries into the open segments or in the time spent in the open segments (Fig. 6C, D). After CNO injection, the treatment group had more entries into the open segments and also spent more time in the open segments than the control group (Fig. 6C, D). The mean speed was similar before and after CNO administration in both the treatment and control groups (Fig. 6E). This result suggests that this one intervention, inhibition of NAc PV neurons using DREADD, mimics the abnormal risk-avoidance behavior of *DISC1-N*^*TM*^ mice.

## Discussion

In this study, we tested risk-avoidance behavior in *DISC1-N*^*TM*^ mice and found an impairment on the EPM test. This test is an approach-avoidance conflict task where anxious mice tend to avoid the open arms and favor the closed arms (‘safe’ zones) more than non-anxious mice [21, 22]. Rodents have a natural aversion for open and elevated areas, as well as natural spontaneous exploratory behavior in novel environments. The EPM relies on rodents' preference for closed spaces (approach) over open spaces (avoidance) [42] and has been directly used to determine risk-avoidance behavior [43]. We also used an elevated zero maze to determine whether inhibition of NAc^PV^ neurons can mimic the abnormal risk-avoidance behavior seen in *DISC1-N*^*TM*^ mice. The elevated zero maze, a modification of the EPM, has the advantage of lacking the scoring ambiguity in the EPM central area. When NAc^PV^ neurons in PV-Cre mice were inhibited, mice spent more time in the open segments than the controls, and also entered these segments more often, indicating an impairment in avoiding potential risks. This suggests that NAc^PV^ neurons are indispensable for, and are able to induce, abnormal risk-avoidance behavior in *DISC1-N*^*TM*^ mice.

To determine which brain regions are involved in the risk-avoidance behavior during the EPM test in *DISC1-N*^*TM*^ mice, we estimated the neuronal activity in 8 regions using c-Fos as an index. We found three regions where c-Fos expression was significantly higher than in WT mice. The NAc, BLA, and VTA all had elevated c-Fos expression (in *DISC1-N*^*TM*^ mice, compared with WT mice), suggesting that these regions are involved in risk avoidance. In a structural investigation of another mutant *DISC1* mouse strain, the NAc expressed an abnormal D2R profile and MSNs had a reduced dendritic spine density [44]. In addition, methamphetamine-induced dopamine (DA) release is significantly potentiated in the NAc of mutant *DISC1* mice [45], and the homeostasis of coincident signaling of DA and glutamate is altered within the NAc [46]. Considering that these changes in the NAc were due to the mutant *DISC1* gene, we decided to target the NAc region in our investigation of abnormal risk-avoidance behavior in *DISC1-N*^*TM*^ mice. Although *DISC1-N*^*TM*^ mice had elevated c-Fos expression in the NAc after the EPM test, reduced activity of NAc PV neurons was found in our subsequent research. One possible explanation for this may be that the decreased activity of PV neurons leads to less inhibition of other major neurons (MSNs) within the NAc, which results in an increase in c-Fos expression. In the NAc area, PV interneurons comprise only 3% of all neurons [47, 48]. Although the proportion is small, studies have shown that PV neurons influence the activity of MSNs in the NAc, including c-Fos expression [49].

Neurons with the capacity to discharge at high rates are called FS-neurons [50]. We found that FS-neurons in the NAc had lower firing rates in *DISC1-N*^*TM*^ mice than in WT mice following tamoxifen administration. In the NAc area, FS interneurons modulate principal neurons through a powerful and sustained feedforward inhibition [51, 52]. PV-expressing interneurons, which constitute 1% of NAc neurons, have particular firing properties and are classified as FS-neurons [51, 53]. Other interneurons in the NAc that express somatostatin, neuropeptide Y, and neuronal nitric oxide synthase are classified as persistently low-threshold spiking neurons [53]. Although many studies have reported that the FS neurons in the NAc are PV neurons, PV interneurons in the NAc are not homogeneous. One study reported that a proportion of NAc FS-neurons also express cannabinoid receptor 1 (CB1) [54, 55]. These NAc^CB1^ neurons partly overlap with NAc^PV^ neurons (52%) [54]. Whether CB1-expressing FS-neurons have the same function as PV-expressing FS-neurons in the NAc, or whether CB1-expressing FS-neurons can be regarded as a subtype of NAc FS-neurons remains an important question. Considering that the striatal PV-expressing interneurons recorded thus far are FS-neurons [56, 57] and the functional properties of FS-neurons reported thus far are uniform [55, 58, 59], we posit that PV can serve as a reliable marker for FS-neurons in the NAc. Using this guidance, we deduced that the FS-neurons we recorded in the NAc were PV neurons. We further explored the function of NAc^PV^ neurons using *in vitro* whole-cell patch clamp recordings and behavioral tests. We found that the current-stimulated firing rate of NAc^PV^ neurons was lower in *DISC1-N*^*TM*^ mice than in control mice and light-evoked activation of PV neurons was able to rescue the risk-avoidance impairment in *DISC1-N*^*TM*^ mice. Both of these results are evidence for our position regarding PV as a marker of FS neurons. Normally, NAc^PV^ neurons receive excitatory inputs from the same brain areas that project to NAc^MSN^ neurons (95% of all neurons in the NAc) and form functional contacts with NAc^MSN^ neurons [49, 58, 59]. Whether NAc^PV^ neurons function in the same way in the risk-avoidance impairment in *DISC1-N*^*TM*^ mice has not been explored yet.

Although many different *DISC1* models have been generated, the phenotypes have not always been consistent [11, 60], and as such, the influence of the *DISC1* gene on mental disorders remains elusive. As an important hub protein, DISC1 interacts with a number of synaptic and cytoskeletal molecules and modulates many cellular functions, such as synaptic plasticity [61] and neurogenesis [62, 63]. Thus, different mutant models (e.g., the *DISC1*-overexpressing mutation model [64]) may influence DISC1 function to varying degrees and lead to different phenotypes. In this study, we used N-terminal fragment *DISC1* transgenic mice to study risk-avoidance behavior; however, further behavioral experiments are required to determine whether there are any other abnormal behaviors in *DISC1-N*^*TM*^ mice.

In conclusion, we tested *DISC1-N*^*TM*^ mice on the EPM and found that NAc^PV^ FS neurons had a lower firing rate *in vivo.* Through *in vitro* patch clamp, we found reduced excitability of NAc^PV^ neurons in *DISC1-N*^*TM*^ mice. This reduced excitability led to a hyperactive NAc (increased c-Fos expression), which impaired approach-avoidance behavior, including an increase in exploration and diminished risk-avoidance. Optogenetically increasing the activity of NAc^PV^ neurons rescued the risk-avoidance impairment in *DISC1-N*^*TM*^ mice. These findings add to our understanding of the neural circuits that are related to environmental risk signals and their evolutionary significance.

## Supplementary Information

Below is the link to the electronic supplementary material.

Supplementary file1 (PDF 162 kb)
