# Supplementary material for: Reduced Firing of Nucleus Accumbens Parvalbumin Interneurons Impairs Risk Avoidance in DISC1 Transgenic Mice
Source: Neurosci Bull. 2021 Jun 18;37(9):1325–38. doi: 10.1007/s12264-021-00731-7 (PMC8423984; doi:10.1007/s12264-021-00731-7)
Supplement: Supplementary file 1 — Supplementary file1 (PDF 162 kb) [file 12264_2021_731_MOESM1_ESM.pdf]

Supplemental Figure

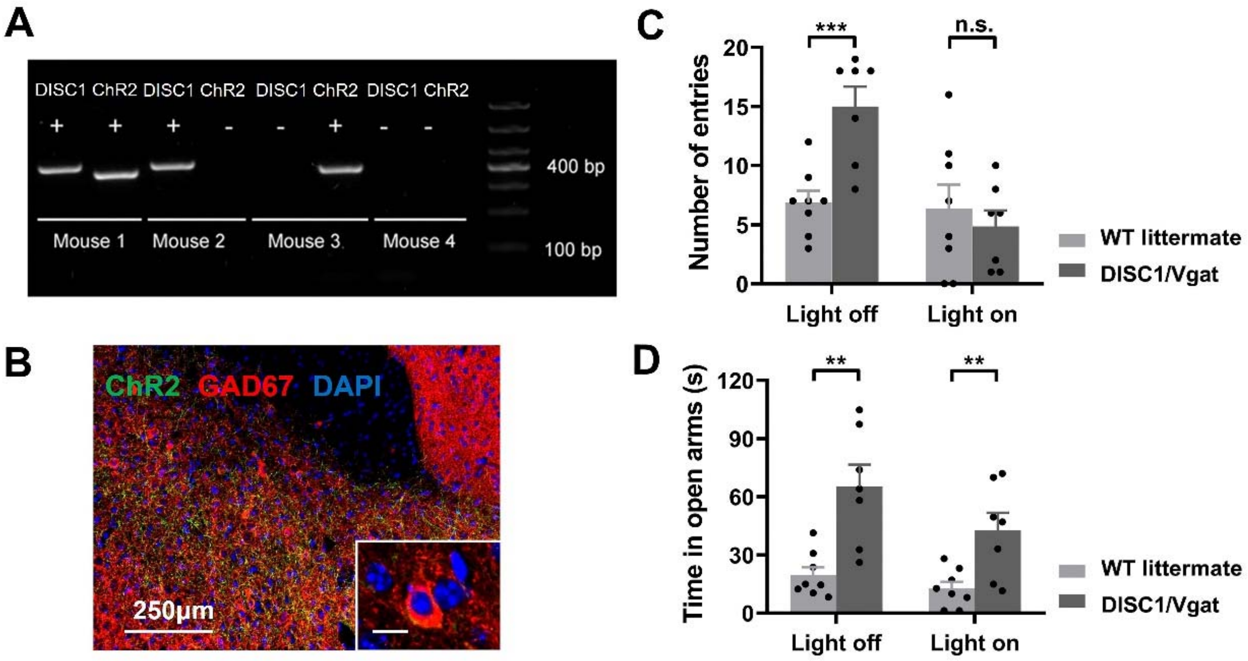

Fig. S1 Optogenetic stimulation of NAc^{GABA} neurons does not fully rescue the risk-avoidance impairment in *DISC1-N^TM^* mice. **A** Sample gel showing the successful generation of DISC1/Vgat-ChR2 double-transgenic mice. **B** Representative confocal image showing targeted ChR2 expression (green) co-stained with GABAergic neurons (red) in the NAc in these double-transgenic mice (scale bar, 250 μm; inset, 10 μm). **C, D** Number of entries (**C**) and time spent in the open arms (**D**) before and after blue light (470 nm) stimulation during the EPM test (unpaired *t*-test, upper ***P = 0.0009, lower **P = 0.0014 (left) and 0.0066 (right); n = 8 left, n = 7 right).
